# Reactive oxygen species during heart regeneration in zebrafish: Lessons for future clinical therapies

**DOI:** 10.1111/wrr.12892

**Published:** 2021-01-20

**Authors:** Olivia Helston, Enrique Amaya

**Affiliations:** ^1^ Division of Cell Matrix Biology and Regenerative Medicine, School of Biological Sciences, Faculty of Biology, Medicine and Health University of Manchester Manchester UK

## Abstract

In humans, myocardial infarction (MI) is associated with irreversible damage to heart tissue, resulting in increased morbidity and mortality in patients. By comparison, the zebrafish (*Danio rerio*) is capable of repairing damaged and injured hearts by activating a full regenerative response. By studying model organisms that can regenerate loss heart tissue following injury, such as the zebrafish, a greater insight will be gained into the molecular pathways that can induce and sustain a regenerative response following injury. There is hope that such information may lead to new treatments or therapies aimed at stimulating a better regenerative response in humans that have suffered heart attacks. Recent findings in zebrafish have highlighted an important role for sustained elevated levels of Reactive Oxygen Species (ROS), including hydrogen peroxide (H_2_O_2_) in the promotion of a regenerative response. Given that elevated levels of H_2_O_2_ can be harmful, simply elevating ROS levels directly may not be easy or practical to translate clinically. An alternative approach would be to identify the critical downstream targets of ROS in the promotion of heart regeneration, and then target these clinically using drugs. One such family of potential downstream targets of ROS during heart regeneration are the family of protein tyrosine phosphatases (PTPs), which are known to be exquisitely sensitive to redox regulation and whose inhibition have been linked to the promotion of heart regeneration in zebrafish. In this review, we present an overview of the zebrafish as a model organism for studying cardiac regeneration, including the molecular mechanisms by which cardiac regeneration occurs in response to injury. We then present recent findings linking elevated ROS levels to heart regeneration and their potential downstream targets, the PTPs, including protein tyrosine phosphatase 1B (PTP1B) and the dual specificity phosphatase 6 (DUSP6) in the promotion of heart regeneration.

## 1 INTRODUCTION

**FIGURE 1:**
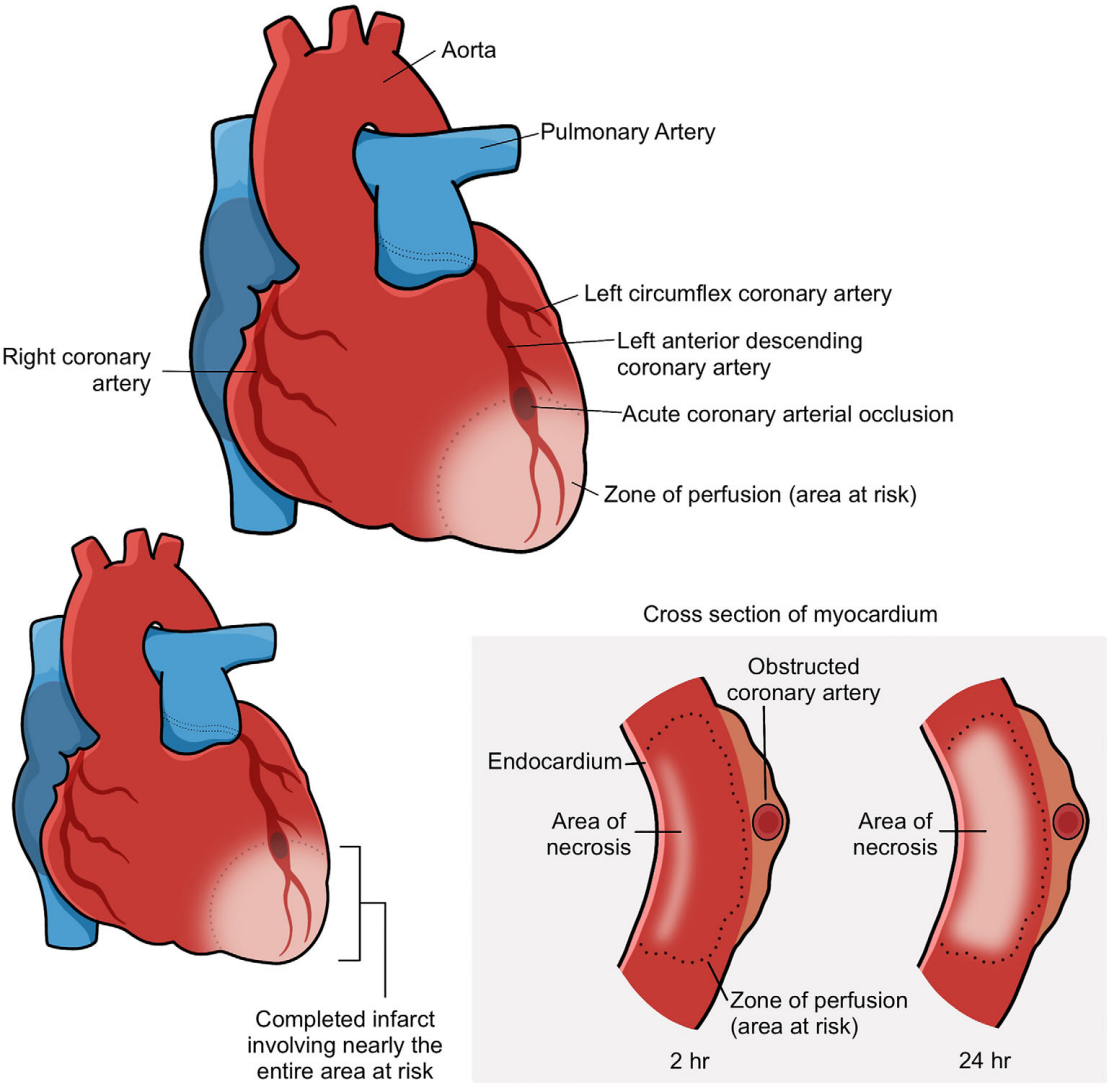
Myocardial infarction. A heart attack occurs when a coronary artery becomes obstructed. This results in progressive loss of myocardial tissue, via loss of perfusion followed by necrosis, distal to the arterial obstruction. Adapted from Kumar et al^5^

The turn of the 20th century saw the charting of a new age in which noncommunicable diseases, including coronary heart disease (CHD), contribute to the majority of lost healthy years of life. CHD remains the greatest cause of mortality worldwide, centring itself as the core of this increasing burden.^1^ CHD is projected to remain the leading cause of mortality worldwide, with 23.3 million people estimated to die from CHDs annually by 2030.^2^ The pertinence of investigating new and successful therapies aimed at treating CHD is thus indisputable. Commonly, CHD is caused by atherosclerosis of the coronary arteries—this sequential endothelial injury, inflammation, and plaque formation results in an inadequate supply of oxygen to the myocardium.^3^ The most common and catastrophic form of CHD is myocardial infarction (MI),^1^ defined as sudden ischaemic death of myocardial tissue.^3^, ^4^ The pathological process of an acute MI consists of intraluminal coronary thrombus formation within an epicardial coronary artery leading to near or total occlusion (Figure 1).^5^ This resultant ischaemia can subsequently lead to ionic and metabolic instability, inducing rapid depression of systolic function of the heart.^4^ Prognosis is extremely variable. While acute MI may cause immediate threat to life in itself, if survived it can contribute to late morbidity and mortality by inducing heart failure (HF)^6^: “a clinical syndrome resulting from any structural or functional cardiac disorder that impairs the ability of the ventricle to fill or eject blood.”^7^ The two remain inextricably linked, with MI remaining the most common cause of HF worldwide.^8^

Current long‐term therapies for MI (and subsequent HF) focus on the alleviation of symptoms associated with decreased cardiac function. These include pharmacological approaches, as well as coronary revascularisation.^9^ As it stands, no finite “cure” for long‐term HF exists other than heart transplantation, which carries a median survival post‐transplantation of a mere 10.7 years.^10^ Graft failure and rejection, infection, and cardiac allograft vasculopathy are some of the severe complications associated with this procedure.^10^

While recent focus has been confined primarily to the development of gene, cell and tissue‐engineering based therapeutics, small molecule regenerative medicine is a developing and emerging area. Owing to their pharmacological control and ease of use, small molecule drugs pose an interesting approach to therapies on a cellular level.^11^

This review will look at the current understanding of the processes behind cardiac regeneration, including the role of reactive oxygen species (ROS), and will focus on zebrafish (*Danio rerio*) as a model system of regeneration. Particular attention will be given to protein tyrosine phosphatases (PTPs) as possible downstream targets of ROS, and potential drug targets for the promotion of heart regeneration.

## 2 DIFFERENCES IN REGENERATIVE CAPACITY WITHIN AND BETWEEN ORGANISMS

Damaged tissue can reach its end state of resolution through two different pathways. Lesions may result in scarring and loss of function or may result in complete restoration of functionality through regeneration. The interspecies variation of such reactions is profound and can also differ between tissues themselves.^12^ Model organisms provide an insight into the molecular pathways of species with high regenerative capacity; this may lead us to determine the differences between such species and poorly regenerating ones such as humans. The zebrafish has positioned itself as one of the most accessible and arguably one of the most important vertebrate models to investigate regenerative mechanisms.^12^ Typically, most vertebrate tissues exert a continuous turnover of cells to maintain homeostasis. This constant cellular turnover is not congruent in all tissues; while we see a high turnover of the keratinocytes of the skin and enterocytes of the gut,^13^ there is little replacement of neurons^14^, ^15^ and cardiomyocytes^16^, ^17^ during adult human life. To put this into perspective, at the age of 25, human cardiomyocytes will renew themselves at an annual rate of 1%. At 75 years old, this becomes a 0.45% turnover.^16^ Among the vertebrates, regenerative capacity is highly variable. Mammals have limited regenerative capacity, restricted to a few organs, such as liver and skin,^12^ or to a short window of time after birth (in the case of the heart).^18^ Some amphibians however have been considered “champions of regeneration” owing to their ability to repair almost any injured body part including limbs, parts of the CNS, and eyes.^19^ The first evidence of adult vertebrate heart regeneration was, in fact, observed through the study of amphibians.^20^

## 3 THE DISCOVERY OF HEART REGENERATION IN ZEBRAFISH

The zebrafish—a modest 2.5–4 cm native of freshwater streams, rivers, and adjacent wetlands in South Asia^21^—has become one of the most widely used vertebrate models for studying developmental and regenerative biology, with hundreds of mutant strains having been produced over the years, including as a result of the Zebrafish Mutation Project.^22^ Adult zebrafish possess the remarkable capacity to regenerate different tissues and organs including the retina,^23^ spinal cord,^24^ fins,^25^ telencephalon,^26^ kidney,^27^ and heart.^28^, ^29^, ^30^ In 2002, Poss and Keating^28^ demonstrated that zebrafish fully regenerate hearts within 2 months after 20% ventricular resection. They concluded that the injured hearts of the zebrafish could overcome scar formation through cardio‐myocyte proliferation, allowing for *de novo* growth of muscle tissue. The study observed that, immediately postinjury, the rapid formation of a fibrin clot prevents the exsanguination of the fish. This clot was then replaced by new myocardium in the following weeks. Remarkably, scar‐less recovery of the ventricular tissue was achieved between 30 and 60 days postinjury.^28^ These findings have stimulated further research using zebrafish as a model organism for dissecting the molecular mechanisms of cardiac regeneration.

### 3.1 Tissue injury models to study heart regeneration

The adult zebrafish heart has a simpler structure than the mammalian heart, containing only one atrium and one ventricle. Despite its anatomical differences, its histological composition is similar to that of other vertebrates.^31^, ^32^ Surgically induced injuries are considered appropriate in the case that whole tissues need to be lesioned, and global regeneration observed. Genetic ablation can be considered where the aim is to observe the regenerative capacity of a particular cell type.^12^

As coronary artery ligation has been precluded as a method of inducing myocardial ischaemia due to the small size of the zebrafish heart, alternative methods have been established, such as ventricular resection, ventricular puncture, cauterization, and cryoinjury.^28^, ^33^, ^34^, ^35^ Cryoinjury, where a metal filament cooled in liquid nitrogen is applied to the ventricular surface of the heart to freeze an area, initiates tissue necrosis through fast freezing and thawing of cells.^35^ The cryoinjury causes the cells surrounding the necrotic area to undergo apoptosis,^35^ and deposition of fibrotic scar tissue via inflammatory cells is seen after the clearance of debris from cell death. Degradation of this large fibrous scar followed by cardiac myogenesis is then observed, and total repopulation by cardiomyocytes occurs within 3–4 months post injury (PI).^36^ These results indicate that scar formation does not repress cardiac regeneration in zebrafish. It is proposed that this method of injury mimics better the pathophysiological processes following an MI in humans than ventricular resection.^36^

### 3.2 Possible explanations of high regenerative capacity in zebrafish

One of the reasons zebrafish may possess a high endogenous regenerative capacity is their ability to reactivate a variety of gene regulatory networks that would otherwise be suppressed in other animal models.^37^ To this end, there has been growing interest in comparing the regenerative capacity present in zebrafish with those exhibited by other teleost fish with sometimes lesser regenerative capacity, such as medaka (*Oryzias latipes*), giant danio (*Danio aequipinnatus*), and surface versus cave subspecies of *Astyanax mexicanus*.^38^, ^39^, ^40^, ^41^ Furthermore, the ploidy of cardiomyocytes has been shown to affect cardiac regeneration. Adult zebrafish cardiomyocytes exist in a mononuclear and diploid state, in contrast to human adults, which are mostly polyploid. Indeed, induction of zebrafish cardiomyocytes to a polyploid state hinders cardiac regeneration.^42^, ^43^

## 4 ORIGIN OF THE REGENERATED CARDIAC TISSUES

### 4.1 Myocardium contributions to heart regeneration

It is important to address the cellular origin of regenerated tissues, as this might influence the design of new therapeutic regenerative strategies. Evidence exists that regenerating cardiomyocytes originate from cardiac progenitor cells. The re‐expression of cardiac progenitor markers including *nkx2.5*, *hand2*, *sox10*, and *tbx20* around the site of damage adds weight to this theory.^44^, ^45^, ^46^ It is also hypothesized that pre‐existing cardiomyocytes surrounding the infarcted area are capable of de‐differentiation. The characteristics of a partially de‐differentiated phenotype include the re‐expression of embryonic myosins, as well as the downregulation of a variety of sarcomeric proteins.^47^, ^48^ Using Cre‐recombinase‐based lineage‐tracing (Cre‐*lox*P), Kikuchi et al^48^ observed that within 1 week of injury, cardiomyocytes contained in the subepicardial ventricular layer express *gata4*—an embryonic cardiogenesis gene. Through Cre‐*lox*P tracing of cells, results showed the constituent cardiomyocytes of the regenerated tissue were derived primarily from pre‐existing myocardium.^47^, ^48^ These findings suggest that reactivation of developmental programs help achieve regeneration.

### 4.2 Nonmuscular contributions to heart regeneration

In addition to the potential involvement of cardiomyocyte progenitor cells and de‐differentiated cardiomyocytes in heart regeneration, other nonmuscle potential contributors have been investigated. Experiments conducted in mice have addressed the potential contribution of epicardial progenitor cells to cardiomyocyte lineage.^49^, ^50^ Inspired by these results, Kikuchi et al^51^ assessed the contribution of epicardial cells during heart regeneration in zebrafish and found that the epicardial cells give rise to perivascular cells, but not to cardiomyocytes or smooth muscle cells. This conclusion was further confirmed by cell transplantation studies, whereby transplanted epicardial cells were seen to infiltrate the infarct and take on the phenotype of perivascular and myo‐fibroblastic cells, but no differentiation into cardiomyocytes was seen.^52^ In the context of the regeneration of endocardium and coronary endothelium, Zhao et al^53^ demonstrated that such tissues derive from pre‐existing endocardial and endothelial cells.

## 5 CELLULAR AND MOLECULAR MECHANISMS PROMOTING CARDIAC REGENERATION IN ZEBRAFISH

The regenerative cycle of the zebrafish heart can be sub‐divided temporally into a number of key phases: (1) Initial inflammatory response, (2) Regeneration of endocardium and epicardium (3) Proliferation of cardiomyocytes, and (4) Fibrotic scar degradation.^30^

### 5.1 Initial inflammatory response

**FIGURE 2:**
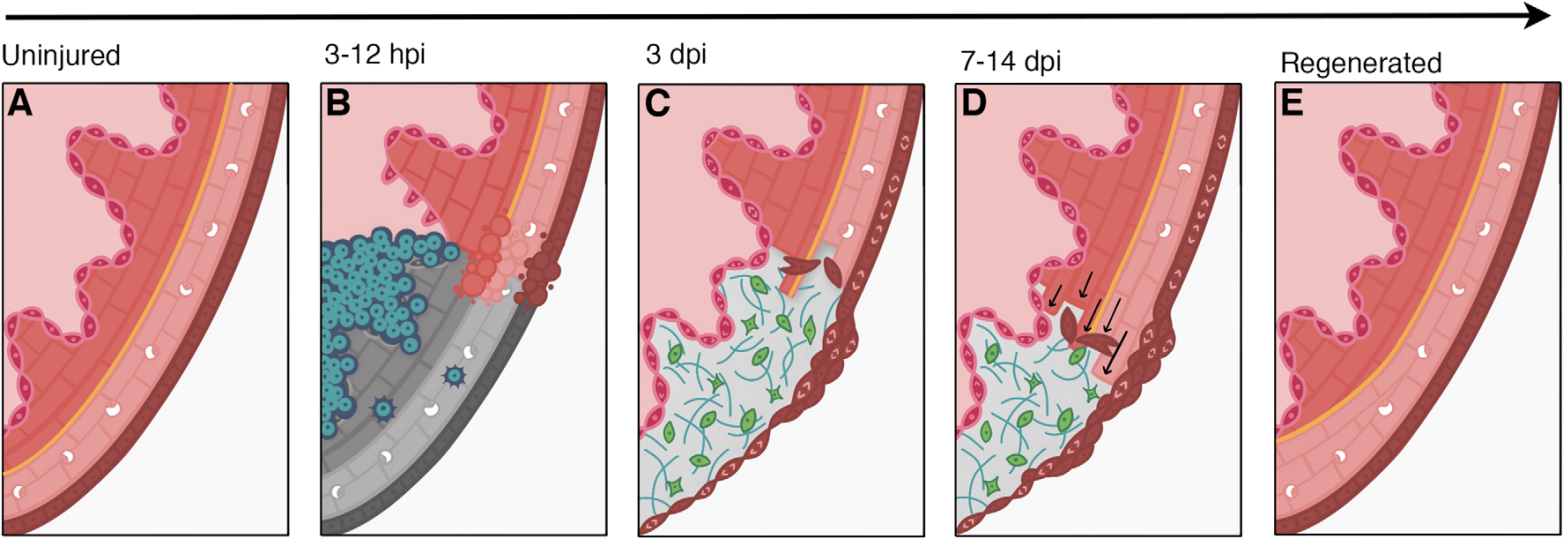
The timeline of events that occur during heart regeneration following cryoinjury in zebrafish. From L‐R (A‐E) A, Cross‐section of the uninjured zebrafish heart showing all three layers of the heart wall. B, Tissue necrosis (gray) induced by cryoinjury in the ventricle. Death of tissue stimulates recruitment of inflammatory cells (blue). C, Epicardial and endocardial cells proliferate and expand over the injured area, D, Cardiomyocyte proliferation and re‐population; as the myocardium regenerates, the fibrotic tissue progressively disappears. E, Advanced stage of regeneration showing restoration of the myocardium

Following injury, initial formation of a fibrin clot protects the fish from exsanguination. The fibrin clot then serves as a site of attraction for myeloid‐derived phagocytes, which can be detected as early as 3 hours postinjury (Figure 2A‐B).^54^ Neutrophils and macrophages then serve as a source of pro‐inflammatory cytokines, including IL‐1β and TNF‐α. These cytokines are not only potent regulators of immune responses but are also responsible for inducing a wave of downstream effects, including initiating angiogenesis and cell proliferation. Huang et al^54^ demonstrated that heart regeneration was impaired in zebrafish following inhibition of IL‐1β and TNF‐α via glucocorticoid treatment. Furthermore, cardiomyocyte proliferation and coronary revascularisation was also impaired when circulating neutrophils and phagocytes were reduced during the inflammatory phase.^54^, ^55^ Through immune response suppression using phagocyte‐targeting liposomes, De Preux‐Charles et al^55^ observed a reduction in mitotic activity, as well as a decrease in fibrin scar degradation at the cryoinjured site. These results suggest that the overall initial immune response is essential for triggering cells to re‐enter the cell cycle and promote heart regeneration.^55^

Alongside the initial inflammation, the epicardium undergoes a dynamic and rapid response to injury. The epicardium is a mesothelial layer surrounding all chambers of the heart, and plays a role in triggering the expression of pro‐regenerative embryonic signals following injury.^56^ By acting as a reservoir of progenitor tissue in embryonic development, the structure serves as a supply of vascular support cells, fibroblasts and possibly cardiomyocytes.^44^, ^56^ In the short window following injury, epicardial tissue proliferates, triggering the expression of embryonic markers including the retinoic acid (RA) synthesizing enzyme, Raldh2.^47^ Initially, the response is seen in all cells of the epicardium.^56^ However, signal expression is quickly localized to the site of injury. To test the role of RA in cardiac regeneration, Kikuchi et al^57^ created two transgenic lines that with inducible impaired retinoid signaling. Histological analysis on the collected ventricles exhibited significantly lower cardiomyocyte proliferation; 68% less than wild‐type ventricles, suggesting RA signaling is needed for myocardial regeneration.

Cytokines released by the activated endocardium (including il11a and il11b) also trigger proliferation of cardiomyocytes. The release of these factors concomitantly activate downstream mediators of the Jak1/Stat3 pathway, which promote the release of Relaxin3a (R1n3a), a stimulating factor of cardiomyocyte proliferation.^58^ Indeed, the Jak1/Stat3 pathway is required for cardiomyocyte proliferation and for heart regeneration.^58^ Regeneration of cardiomyocytes can also be permanently blocked by inducible overexpression of a dominant negative *vegfaa* (vascular endothelial growth factor A‐A) construct.^49^ Marin‐Juez et al^59^ concluded that early vascularisation following cryoinjury is dependent on *vegfaa* and they proposed that revascularisation was an early‐stage event, with evidence of sprouting angiogenesis as early as 15 hours PI at the site of the lesion. This suggests that prompt and sufficient revascularisation is a crucial factor for promoting cardiac regeneration.

### 5.2 Regeneration of the endocardium and epicardium

Regeneration of the nonmuscular tissues of the heart occurs before the myocardium and is often completed within the first few days PI.^60^, ^61^ Endocardium surrounding the lesion site proliferate between 3 and 5 days PI (Figure 2C *)*. These events precede the peak rate of cardiomyocyte proliferation, which follows at 7 days PI.^60^, ^61^ (Figure 2D *)*. Endocardial proliferation is dependent on Notch signaling, and the expression of *serpine1*.^60^, ^62^, ^63^, ^64^ One study conducted by Munch et al^60^ demonstrated a reduced number of endocardial cells and an increased amount of fibrotic tissue at the site of injury when Notch signaling is inhibited. Serpine1 acts as an early endocardial injury‐response factor and its downregulation is dependent on Notch. Zebrafish treated with a *serpine1* inhibitor expressed a number of augmented endocardial cells surrounding the wound site, suggesting that endocardial proliferation is linked to the downregulation of *serpine1*.^60^ Furthermore, Notch signaling promotes cardiomyocyte regeneration by dampening downstream myocardial Wnt activity following injury.^53^ Zhao et al^65^ observed that endocardial expressed Notch upregulates the expression of secreted Wnt antagonists *notum1b* and *wif1*. The expression of these antagonists are beneficial, as increased Wnt signaling is associated with regenerative failures. Taken together, this evidence suggests a potential angle for promoting endo and epicardial regeneration through augmentation of the Notch signaling pathway.

The next stage of regeneration involves the migration of epicardial cells over the lesioned area shortly after injury; beginning at the base of the heart and continuing toward the apex.^66^ (Figure 2D). During embryogenesis, the epicardium migrates out of a cluster of mesodermal derived cells and envelops the developing myocardial tube. Following this formation, some of the epicardial originating cells undergo an epithelial‐to‐mesenchymal transition (EMT) and infiltrate the myocardium^67^ These cells form the endothelium and smooth muscle contained in the coronary vasculature.^68^ Via the induction of the ligand Fgf17b in the myocardium, and the Fgfr2 and Fgfr4 receptors induced in adjacent epicardial derived cells, coronary neovascularisation is promoted.^44^ Interestingly, a similar series of events occur following injury, as epicardial cells around the wound margin reactivate the expression embryonic genes (including *raldh2*, *tbx18*, and *wt1b*), begin proliferating and undergo EMT, thus becoming epicardial‐derived cells (EPDCs), which later generate smooth muscle and endothelial cells of the coronary vasculature.^36^ Injured epicardial cells also undergo a series of extensive morphological changes: normal epicardial cells possess a flattened, classic epithelial morphology; however following cryoinjury, cells loose cellular adhesions.^52^ In addition, fibroblast growth factors (Fgfs) are secreted by surrounding cardiomyocytes, while thrombocytes secrete platelet‐derived growth factors (Pdgf) to induce EMT and the formation of EPDCs. These growth factor pathways are essential for correct epicardial function and neovascularisation, as their inhibition result in poor revascularisation and impaired regeneration.^44^, ^69^

Hedgehog signaling (Hh) also plays a key role in controlling epicardial migration. Wang et al^66^ demonstrated ex vivo that removal of the bulbous arteriosus (BA), disrupts epicardial regeneration. This is due to the expression of Hh ligand in the BA, as inhibiting Hh signaling is sufficient to arrest epicardial regeneration.^66^ The transcription factor gene *tcf21+* is a marker that displays epicardial‐specific expression throughout the development and regeneration of cells.^51^ Through the genetic ablation off *tcf21+* cells, Wang et al^66^ demonstrated a reduction in cardiomyocyte proliferation and regeneration. From this evidence it can be inferred that Hh signaling is essential for promoting epicardial migration and thus the significant regenerative capacity of ventricular epicardial cells.

### 5.3 Proliferation of cardiomyocytes

Cardiomyocyte proliferation is induced through paracrine signals from nonmuscle cells. One of these paracrine signals is TGFβ signaling. TGFβ plays a critical role in the pathogenesis of cardiac remodeling and fibrosis.^70^, ^71^ Overexpression of TGFβ results in excessive cardiac fibrosis and hypertrophy.^70^ Targets of this pathway include extracellular matrix (ECM) proteins, such as Fibronectin, Tenascin C, and Collagen.^72^ Prolonged and extended inhibition of this pathway results in impaired cardiomyocyte proliferation^72^ Importantly, infiltration of de novo cardiomyocytes into the area of infarct is accompanied by a simultaneous regression in the local fibrous scar tissue. It can therefore be inferred that TGFβ may serve a role as a key regulator for the transition of the infarcted tissue to the formation of the scar. The mechanism behind this transition may lie in TGFβ's ability to deactivate inflammatory macrophages while also promoting the formation of myofibroblasts and their synthesis of ECM proteins.^70^, ^73^

Additional extracellular signals that cardiomyocytes are exposed to include insulin growth factor (Igf), bone morphogenic protein (BMP), and Neuregulin 1 (Nrg1). Studies have demonstrated the importance of Igf signaling in murine embryological cardiomyocyte proliferation.^74^ Using evidence from microarray‐based gene expression analysis, Huang et al^75^ performed a study testing the role of this signal after ventricle resection in zebrafish. Results showed that Igf levels peaked between 3 and 10 dpa and was expressed in all areas of the wound. This trend is concordant with a functional role of Igf signaling in regeneration, given that cardiomyocyte proliferation peaks between 7 and14 dpa.^28^, ^74^ (Figure 2D,E). The literature also demonstrates there is an embryonic role for Igf, as expression is also observed in both the developing mouse^74^ and zebrafish^76^ hearts. This suggests that there is a reactivation of developmental genes that allows for the maintenance of high regenerative capacity within zebrafish.

Molecular regulation of cardiomyocyte regeneration can also be attributed to the BMP signaling pathway.^77^ While there is plenty of evidence supporting the role of BMP signaling in cardiovascular development,^78^ there has been conflicting evidence for its role in cardiac regeneration. While BMP2 protein has been shown to induce the re‐entry of cardiomyocytes into the cell cycle as well as reduce the rates of apoptosis,^79^, ^80^, ^81^ BMP4 protein has been observed to enhance apoptosis of cultured cardiomyocytes.^82^ Wu et al^83^ observed that among the genes enriched at the site of the injury included BMP pathway genes. Interestingly, the authors concluded that BMP signaling played a regulatory role only during cardiomyocyte proliferation in the instance of regeneration but does not contribute to the regulation of cardiomyocyte proliferation during cardiogenesis.^83^

The expression of Nrg1 has multiple influences in cardiovascular biology but possesses a key role in contributing to normal cardiomyocyte development.^84^ Nrg1 is a member of the epidermal growth factor (EGF) family and is essential for normal heart morphogenesis during development in both zebrafish and mice, and plays a key role in the process of cardiac myofiber trabeculation. Murine studies observed multiple physiological cardiac abnormalities in animals that carried neuregulin mutations or carried mutations in its receptors, ErbB2 or ErbB4.^85^, ^86^, ^87^ In addition, histological analysis confirmed poorly developed ventricular trabecules; an anatomical structure derived from cardiomyocytes.^84^ Bersell et al^88^ discovered that upon systemic injection of Nrg1 there was a small, but measurable improvement in cardiac repair after MI, although quite small. Gemberling et al^89^ thus decided to investigate the role of Nrg1 in zebrafish. Through small molecule inhibition of Nrg1 receptors (Erbb2 and Erbb4b), they noted a 54% decrease in cardiomyocyte proliferation following injury.^89^ This indicates that Nrg1 signaling is essential for successful heart regeneration.

### 5.4 Fibrotic scar degradation

The initial reaction of ECM deposition after injury changes, and in the weeks following, regenerated cardiomyocytes replace the ECM at the lesion site^90^ (Figure 2F). Macrophages are the most abundant type of inflammatory cell during scar resolution.^91^ One gene highlighted as a downstream target of the inflammatory response following injury is *spp1*, which encodes Osteopontin (Opn).^92^, ^93^, ^94^ Bevan et al^90^ investigated the role of Opn in regeneration in zebrafish. Interestingly, the authors observed the existence of two distinct phases of macrophage populations: tnfα+ and tnfα−. These waves exist at two different points after injury, and augmentation of these waves results in significant impairment in the production and subsequent resolution of scar material. By creating *opn* mutant lines, they were able to observe that Opn possesses a pivotal role in the change in macrophage phenotype (at around 3–7 days post injury), from tnfα+ to tnfα−. Mutant lines possessed reduced Collagen I deposition at 3–7 days PI, however, the amount of scarring was increased at later points in the animals' recovery. From their results they concluded that Opn possesses a bi‐functional role and contributes to both the deposition and resolution of the scar.^90^ This study demonstrated similarities in the inflammatory and scar‐deposition stages in mammals and zebrafish. It highlighted that the inflammatory stage was rapidly resolved, and scar formation was transient in the latter and demonstrates a critical role for macrophages in regenerative, as opposed to fibrotic cardiac repair.

## 6 THE ROLE OF ROS IN CARDIAC REGENERATION

In recent years focus has turned toward investigating the molecular mechanisms that initiate a regenerative response, following injury. During regeneration there lies a series of conserved events across many organisms. Immediately following injury, the wound surface is closed by the movement of surrounding epithelial cells, which subsequently thickens to form the wound epithelium. Under this, cells migrate to form a blastema—a tightly packed mass of proliferative cells. Following this, differentiation and proliferation occurs. One of the key questions to be asked is how the process of tissue damage sets in motion a regenerative response? The initial insult of injury causes the release of a number of signals including ATP, calcium and importantly, ROS.^95^, ^96^, ^97^, ^98^ The extensive literature surrounding the role of sustained ROS production during appendage regeneration across various organisms may help guide our efforts to understand the role of ROS in heart regeneration.

### 6.1 Reactive oxygen species

By definition, ROS are highly reactive ions and free radicals; a product of the partial reduction of oxygen. They can exist in multiple forms, including hydrogen peroxide (H_2_O_2_), the superoxide anion (O_2_^−^), and the hydroxyl radical (OH^−^). Owing to their reactivity, they tend to be particularly unstable, and have a short half‐life. Their production is a result of many biological and biochemical processes or from exposure to radiation or toxic components.^99^ In eukaryotic cells, the mitochondrial electron transport chain is a major source of intracellular ROS production. During this reaction, the superoxide anion is generated at complex I, II, and III, via a precocious one‐electron reduction of molecular oxygen.^100^, ^101^ Currently, little is known about the mechanisms that regulate mitochondrial ROS production. However, it has been hypothesized that proton‐motive force may be of influence. This force is the combined effects of the electrical and pH gradients across the inner mitochondrial membrane. An increase in proton motive force has a positive effect on ROS production.^102^ Mitochondrial ROS production is also thought to be regulated by the surrounding availability of oxygen. Interestingly, both hypoxic and hyperoxic conditions have been shown to increase intracellular ROS levels.^103^

One proposed explanation for the reduced proliferative capacity of adult mammalian cardiomyocytes is oxidative DNA damage. For a short period of time after birth, the mammalian heart possesses a remarkable regenerative capacity.^104^ Afterward, a series of postnatal events triggers cardiomyocytes to permanently exit the cell cycle. Using the murine model, Puente et al^98^ demonstrated that DNA damage as a result of mitochondrial ROS can trigger cell cycle arrest in adult cardiomyocytes. They hypothesized that the exit of cardiomyocytes from the cell cycle was a trade‐off in exchange for the sudden switch to oxygen‐dependent aerobic metabolism required in the oxygen‐rich postnatal environment. Their results showed a significant increase in the levels of ROS, oxidative DNA damage and DNA damage response (DDR) markers in the heart in the first week after birth. These finding suggest that hypoxia and redox signaling could be important contributors to the regulation of cardiac proliferation. Indeed, exposure to systemic hypoxaemia in adult mice results in decreased ROS production in the heart and a promotion of cardiomyocyte proliferation and regeneration following injury.^105^ Thus, for immature and/or mature myocytes or progenitor populations to proliferate effectively, a lower oxygen concentration is needed.^106^ Interestingly, metabolic reprogramming that promotes glycolysis at the expense of OXPHOS has been previously proposed to be an important aspect of tissue and appendage regeneration.^107^ Indeed, heart regeneration in zebrafish was recently shown to be associated with enhanced glycolysis and diminished OXPHOS, suggesting that such metabolic reprogramming is essential for heart regeneration as well.^108^, ^109^

### 6.2 Duox, Nox, and heart regeneration

In addition to mitochondria, another major source of cellular ROS production are the NADPH oxidase family of enzymes (NOXes).^110^ Existing largely as multi‐subunit enzymes comprising of membrane and cytosolic components, NOXes participate in a variety of cellular functions, including host cell defense, gene expression regulation, and cellular signaling.^111^ The NOXes produce superoxide anions by catalyzing the transfer of a single electron from the electron donor NADPH to molecular oxygen.^112^ Subsequently, via the rapid process of spontaneous dismutation, or catalytic reactions via Cu^2+^Zn^2+^‐superoxide dismutase, H_2_O_2_ is formed.^112^ To investigate the role of ROS in heart regeneration in zebrafish, Han et al^113^ identified a number of NADPH oxidase genes, which are elevated during heart regeneration, including several genes encoding subunits in the Nox2 complex. In addition, *duox*, another gene enconding an NADPH oxidase family member, was also upregulated especially in the epicardium during heart regeneration starting from 3 days post injury.

Unlike the rapid rise of H_2_O_2_ concentration and subsequent inflammatory response seen in zebrafish following tail fin amputation,^95^, ^96^ H_2_O_2_ production following ventricular resection follows a slower timecourse, with no detectable increase at 1‐hour post injury.^113^ Instead, H_2_O_2_ was primarily elevated from 3 days onward post injury in the epicardium and adjacent myocardium of the proximal resection site, which strongly correlates to the areas of myocardial regeneration.^113^

Han et al^113^ sought to establish the role of Duox/Nox2 and H_2_O_2_ production during heart regeneration by attenuating ROS levels during heart regeneration using a transgenic line with cardiac‐specific catalase or following the application of NADPH oxidase inhibitors. These permutations resulted in fewer regenerated myocytes as well as increased cardiac fibrosis.^113^ Overall their study suggested that elevated levels of H_2_O_2_ is necessary for cardiac regeneration.^113^

## 7 PTPs AS POTENTIAL KEY DOWNSTREAM TARGETS OF ROS

**FIGURE 3:**
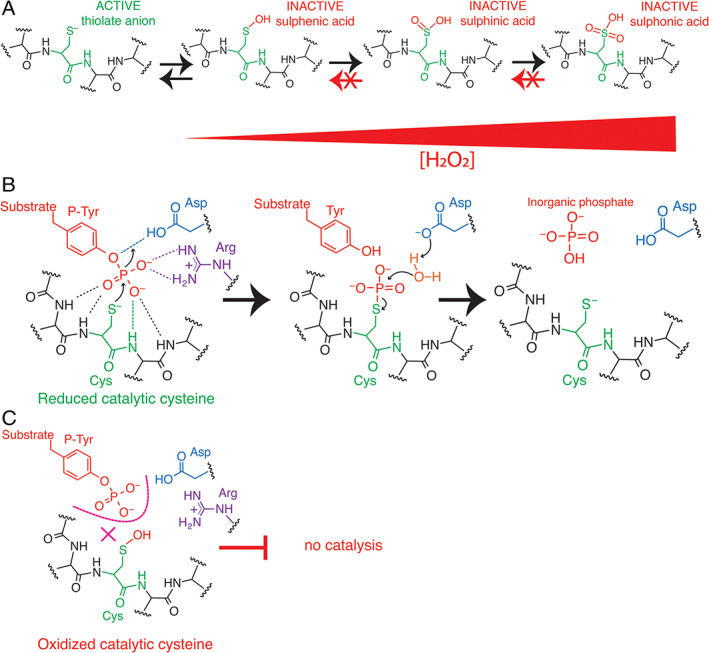
Mechanism of reversible inhibition of protein tyrosine phosphatases via oxidation. A, The thiolate anion (S‐) in cysteine residues in proteins are highly susceptible to oxidation by reactive oxygen species, such as hydrogen peroxide. At low concentrations, H_2_O_2_ will oxidize the thiolate anion to a sulphenic acid (S‐OH) form, which is reversible back to the thiolate anion form as ROS levels fall. However, as H_2_O_2_ levels continue to rise, this results in irreversible oxidation forms, such as the sulphinic (S‐O_2_H) and sulphonic (S‐O_3_H) forms. B, Mechanism of catalysis of protein tyrosine phosphatases (PTPs). Hydrogen bonding (hashed lines) in the catalytic site of PTPs cradle the phospho‐tyrosine residue (red) of the substrate protein. The thiolate anion in the catalytic site attacks the phosphate in the tyrosine, leading to the dephosphorylation of the tyrosine residue, ultimately returning the catalytic cysteine (green) to the thiolate anion form again. The other critical amino acids in the catalytic site, which assist in forming the catalytic site are asparagine (blue) and arginine (purple). C, Inhibition of catalysis of PTPs via oxidation of the catalytic cysteine. When the thiolate anion is oxidized to the sulphenic, sulphinic or sulphonic forms, this inhibits the catalytic activity of the PTPs. When oxidation is only to the sulphenic form, this inhibition is reversible, and PTP activity returns when the catalytic cysteine returns to the thiolate form, as in B

While a critical role for sustained ROS production during tissue regeneration is now recognized as an ancestral/conserved mechanism across metazoans, including planarians, *Drosophila*, zebrafish, amphibians, reptiles, and mammals,^96^, ^97^, ^114^, ^115^, ^116^ little is known about what are the critical downstream targets of elevated ROS production, which promote regeneration. Phosphorylation is a major post‐translational event that regulates many cellular processes, including many of those necessary for tissue regeneration, such as growth factor signaling, cell cycle control, and metabolic reprogramming. Kinases are enzymes that catalyze the phosphorylation of serine/threonine or tyrosine residues. The kinases themselves are often activated via phosphorylation of key residues on the enzymes. The activity of kinases is usually counteracted by those of phosphatases, which keep the activity of kinases in check. They do so by catalyzing the removal of the key phosphate groups, resulting in the suppression of downstream pathways.^117^ One key family of enzymes, the protein tyrosine phosphatases (PTPs), find themselves particularly interesting potential downstream targets of ROS, as they are exquisitely sensitive to redox regulation.^118^, ^119^ This is primarily due to the fact that the catalytic center of PTPs, (I/V)H**C**XXGXXR(S/T), contains a redox sensitive cysteine residue, which is often present *in vivo* as a negatively charged thiolonate anion (Figure 3A). The thiolonate anions in the catalytic center of PTPs makes them particularly sensitive to the presence of cellular oxidants, including H_2_O_2_.^119^ Importantly, while high concentrations of H_2_O_2_ can oxidize the catalytic cysteine of PTPs to the nonreversible sulphinic (SO_2_H) and sulphonic (SO_3_H) forms, lower levels of H_2_O_2_ will oxidize the catalytic cysteine of PTPs to the reversible sulphenic (S‐OH) form (Figure 3A). The mechanism of catalysis of PTPs require the catalytic cysteine to be present in the thiolonate anion form, where it can act as a strong nucleophile for phosphorylated tyrosine residues in protein substrates (Figure 3B).^118^ When oxidized, even to the sulphenic form, PTPs become catalytically inactive (Figure 3C). Given that oxidation to the sulphenic form is reversible, rising and lowering ROS levels are able to reversably switch PTP activity on and off, thereby regulating a number of signaling pathways involving tyrosine kinases and phosphatases. In addition, it is notable that there are more than 100 members in the PTP superfamily of enzymes in the vertebrate genome,^120^ with most of them sharing the signature catalytic center containing cysteine, making them possible downstream targets of ROS regulation. Two members of the PTP family that have already been linked to heart regeneration are Dusp6 and Ptp1b.

**FIGURE 4:**
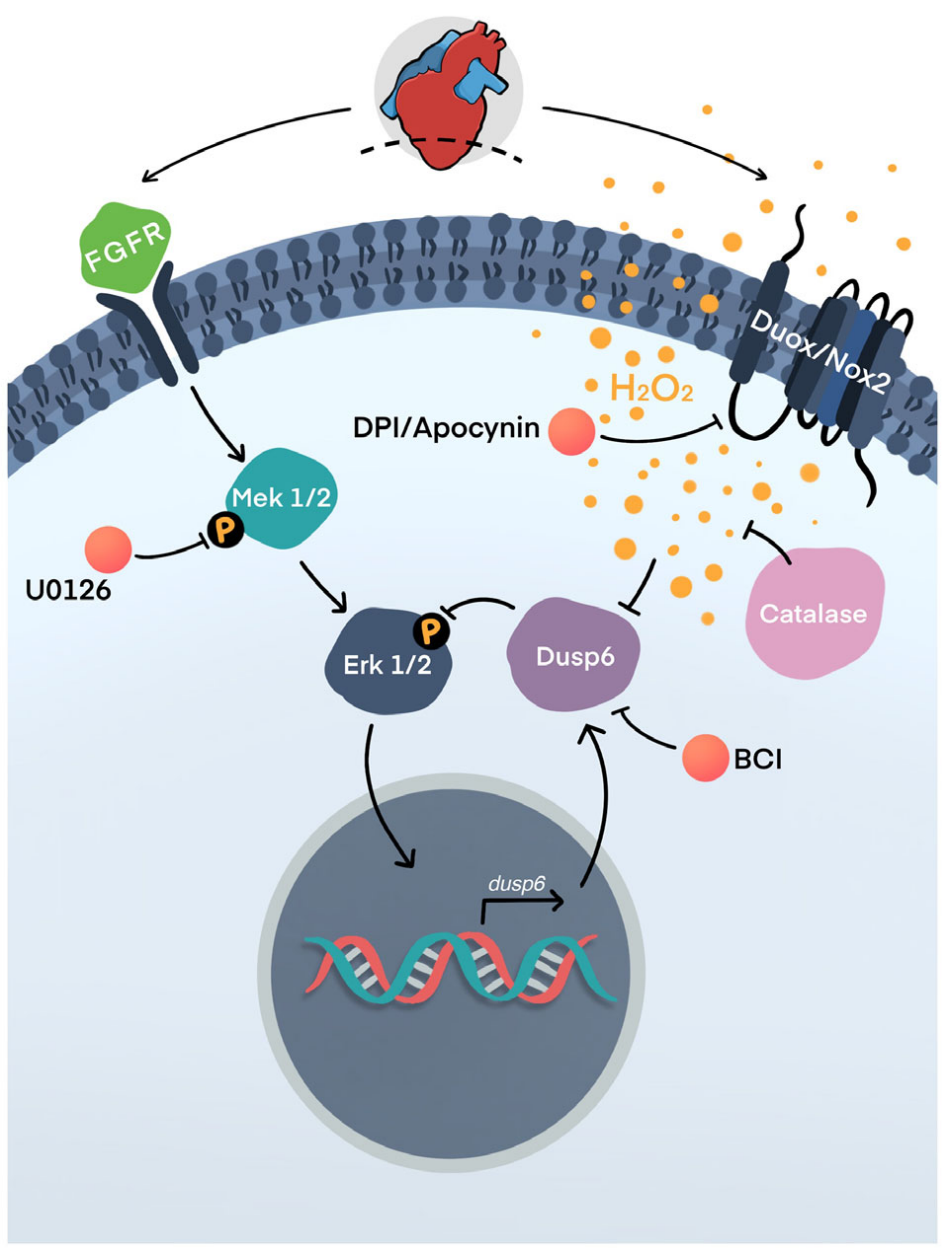
Reactive oxygen species/hydrogen peroxide promotes zebrafish heart regeneration via a de‐repression mechanism involving the Dusp6. Upon injury, growth factor signaling activates the Ras/MAPK pathway. The activation, via phosphorylation, of Mek1/2 and Erk1/2 induces expression of *dusp6*. Dusp6 in turn dephosphorylates ERK1/2, thus de‐activating the FGF/Ras/MAPK pathway, which is necessary for heart regeneration. However, injury also stimulates H_2_O_2_ production close to the site of injury site via the activation of Duox and Nox2. The elevated levels of H_2_O_2_ reversibly inhibits the Dusp6 phosphatase via the oxidation of its catalytic cysteine, thus sustaining Ras/MAPK activation. Thus, the production of H_2_O_2_ promotes heart regeneration by a de‐repression mechanism. DPI and apocynin are inhibitors of Duox/Nox2. Catalase is an enzyme that destroys H_2_O_2_. CI is an inhibitor of Dusp6. U0126 is an inhibitor of Mek1/2. Adapted from Han et al^113^

### 7.1 The Duox H_2_O_2_ pathway and Dusp6

As previously discussed, growth factor signaling pathways such as FGF and PDGF are essential for successful adult heart regeneration.^44^, ^69^ Downstream of these developmental growth factors pathways are the Ras/MAP kinase pathway (also known as ERK pathway).^121^ Interestingly phosphorylation of Erk1/2 (pErk) is elevated in lesioned hearts at 3, 7, and 14 days post injury, with pERk1/2+ cells localized primarily to the epicardium.^113^ Furthermore, decreasing Erk1/2 phosphorylation using an inhibitor compromises regeneration of myocardial cells. Furthermore, the generation of Gata4‐EGFP+ myocardial cells is also diminished when the NADPH oxidases, Duox and Nox2 are inhibited.^113^ These results suggest a link between epicardial Erk1/2 activation and H_2_O_2_ production during cardiac regeneration in the adult zebrafish.

Erk1/2 phosphorylation state is negatively regulated by the dual specificity phosphatase 6 (Dusp6), a member of the PTP superfamily, which is sensitive to redox regulation by ROS.^122^ Dusp6 exists during embryonic development as a potent feedback attenuator of MAPK activation and functions, in part, to dampen FGF‐stimulated MAPK signaling.^121^, ^123^ Dusp6 expression is also seen in both endothelial cells and cardiomyocytes of the adult zebrafish heart following ventricular resection.^122^ Furthermore, *dusp6* expression is induced within 1 day after injury, and its elevated expression continues to 7 dpa, in correlation with the initiation of regenerative events. This localization suggests Dusp6 might play a crucial role in the early stages of cardiac regeneration. Missinato et al^122^ demonstrated that *dusp6* mutant fish were normal during early development but went on to demonstrate thicker and more compact myocardium, with increased number of blood vessels than the wildtype hearts.

Since Dusp6 suppresses ERK activity, researchers sought to ask how the loss of *dusp6* alters cardiac regeneration? *Dusp6* mutants exhibited a significant increase in the number of proliferating cardiomyocytes following injury, however this was not an indefinite effect, with results showing proliferation of the cardiomyocytes was limited to the first 10 days postinjury.^122^ This suggested that diminishing Dusp6 activity may improve cardiac regenerative capacity. Importantly, the *dusp6* mutants developed normally, exhibiting no embryonic or adult lethality. This suggests that *dusp6* is not essential for embryonic development, or that its absence during development can be compensated by other Dusp family members.^122^ In this instance, Dusp2, Dusp4, Dusp5, and Dusp7 can also dephosphorylate Erk1/2 and could thus compensate for the loss of Dusp6.^124^, ^125^

Dusp6 is also a redox‐sensitive target of H_2_O_2_. On one hand, *dusp6* expression is increased by Erk1/2 activation, but reciprocally Dusp6 dephosphorylates and deactivates Erk1/2. This negative feedback loop forms a limiting cycle. However, H_2_O_2_ has the ability to reversibly oxidize Dusp6, breaking the negative feedback loop, resulting increased and/or sustained activation of pro‐regenerative Erk1/2 signaling.^113^ This process is summarized in Figure 4.

In summary, H_2_O_2_ likely produced by Duox/Nox2 reversibly oxidizes and inhibits Dusp6 to impart a de‐repression mechanism by disengaging the negative feedback between Dusp6 and Erk1/2. The ability of the enzyme to switch from an active to an inactive state by simply augmenting ROS levels make it a strong potential target of ROS during regeneration.

Importantly, and perhaps more relevant to the development of clinical therapies and use in humans, one could consider using chemical inhibitors with greater specificity than ROS to achieve reversible regulation of PTPs, such as Dusp6 to promote heart regeneration in the clinic. Using BCI and BCI215, which directly inhibit the phosphatase activity of Dusp6, Missinato and colleagues^122^ were able to observe an increase in cardiomyocyte proliferation and angiogenesis and a reduction in fibrosis after cardiac injury. The increased rate of proliferation was observed during 4–7 dpa and stopped at 12 dpa; this is in line with the time period when Dusp6 functions to attenuate the Ras/MAPK pathway.^122^

### 7.2 Ptp1b—another key potential downstream target of ROS

Another PTP family member that possesses similar reversible redox regulation to Dusp6 is the Protein tyrosine phosphatase 1b (Ptp1b), the product of the *ptpn1* gene. While Dusp6 is a dual specificity phosphatase, Ptp1b is a nonreceptor protein tyrosine phosphatase. Ptp1b has garnered particular interest as it has been shown to modulate a number of metabolic pathways, including insulin and leptin signaling.^126^ Like Dusp6, ROS have the potential to reversibly inactivate Ptp1b.^127^ These similarities have led researchers to investigate the role of Ptp1b in cardiac function, including its inhibition as a potential approach to promoting cardiac regeneration.

### 7.3 Ptp1b inhibition using small molecules to promote cardiac regeneration

Unlike other therapeutic strategies, small molecule therapies are often less complex, and have fewer regulatory hurdles associated with their production and implementation.^128^, ^129^ Furthermore, they are often reversible, and potentially more cost‐effective.^128^, ^129^

One study conducted by Smith et al^130^ set out to identify small molecules that could promote innate tissue repair and regeneration processes in zebrafish. Their phenotypic screen identified one naturally occurring small molecule: MSI‐1436 (also known as trodusquemine), an aminosterol derived from the liver of the dogfish shark,^131^ that selectively inhibits Ptp1b.^132^, ^133^ Their results showed that intraperitoneal injections of MSI‐1436 increased the number of proliferating cardiomyocyte cells by 2.6 fold following partial ventricular resection. In vivo‐morpholino knockdown of Ptp1b in adult zebrafish revealed similar effects to MSI‐1436 injections on cardiomyocyte proliferation.^130^ Smith and colleagues also analyzed the effect of MSI‐1436 on the rate of overt heart regeneration in zebrafish by analyzing the expression of tropomyosin—a marker specific to muscle and expressed by cardiac sarcomeres. Results revealed an almost two‐fold increase in tropomyosin expression within the injury zone at 21 dpa.^130^ Given that the regeneration process of the adult zebrafish heart is complete within 2 months,^28^ these results suggest that MSI‐1436 may be a potent stimulator of cardiomyocyte proliferation and the subsequent regeneration of new heart muscle. In addition, MSI‐1436 is also capable of increasing the rate of caudal fin regeneration in adult zebrafish—a structure comprised of multiple tissues and cell types.^130^ This suggests that MSI‐1436 may accelerate the regeneration process of multiple tissue types and organs. Due to the ubiquitous nature of Ptp1b and its function in dephosphorylating and inactivating receptor tyrosine kinases, it is difficult to pinpoint the exact cell types and processes it is acting upon. From the above results, however, we may expect that inhibition of Ptp1b enhances the activity of multiple pro‐regenerative signaling pathways. In addition, given the exquisite, yet reversible, redox sensitivity of PTP1B to elevated ROS levels,^127^ one may also expect that Ptp1b may be an additional critical downstream target of ROS production during heart regeneration.

### 7.4 Developments in Ptp1b inhibitors

Ptp1b inhibits leptin and insulin‐dependent pathways, making it a promising target for some metabolic pathologies.^126^ Leptin functions to control appetite and energy expenditure, while insulin regulates glucose uptake. Over the past decade, Ptp1b inhibitors, such as MSI‐1436, have been tested in phase 1 trials for obesity and diabetes.^117^ This allows for the evaluation of its safety efficacy as a small molecule inhibitor for use in humans. Its success in these trials with the absence of any adverse events or observations has opened the door for its application as a therapy for targeting other pathologies, such as cardiac disease. One draw‐back observed however is its lack of oral bioavailability, with current drug delivery completed via injections—a poor option for long term treatment.^117^ While Ptp1b null mice are protected against cardiac dysfunction, and ultimately HF following MI, one study obtained similar results using another pharmacological inhibitor AS279.^134^ Many studies have focused on the role of MSI‐1436 and other similar Ptp1b inhibitors in the prevention of cardiovascular disease through the reduction in formation of atheromatous plaques.^135^ While ultimately this may lead to a reduction in the progression to cardiac insufficiency, less has been done to investigate the potential of Ptp1b inhibitors in promoting regeneration following acute ischaemia. No pharmacological inhibitors have yet been tested for this application, however early trials in other areas have shown promising results, with the next generation of Ptp1b inhibitors well on its way.^136^

## 8 CONCLUSION

The stimulation of multi‐tissue and organ regeneration through the inhibition of PTPs as seen in zebrafish and murine models is promising. The ability to stimulate regeneration of tissues and organs, including the heart, without inducing tissue malformation place small molecule inhibitors of PTP family members, such as MSI‐1436 and BCI, as a highly promising pharmaceutical approach aimed at addressing a wide variety of diseases, whose burdens on current healthcare systems are only increasing as a result of modern lifestyles. While there has been progress in large animal studies looking at the use of pluripotent‐stem‐cell‐derived cardiomyocytes and direct reprogramming in promoting cardiac regeneration,^137^ many barriers still exist in translating their use into the clinic. In particular, some remain fearful at the potential of inducing neoplastic transformation.^137^ As an alternative, one should not lose sight of the great potential of small molecule therapeutics aimed at stimulating a regenerative response in endogenous tissues and organs.

## CONFLICT OF INTEREST

The authors declare that they have no conflict of interest for this article.
